# Validation of the neuropathological criteria of the fourth Consortium on dementia with Lewy Bodies in autopsy cases from psychiatric hospitals

**DOI:** 10.1111/pcn.13814

**Published:** 2025-03-31

**Authors:** Kazuhiro Takeda, Hiroshige Fujishiro, Youta Torii, Hirotaka Sekiguchi, Shusei Arafuka, Chikako Habuchi, Ayako Miwa, Norio Ozaki, Mari Yoshida, Shuji Iritani, Yasushi Iwasaki, Masashi Ikeda

**Affiliations:** ^1^ Department of Psychiatry Nagoya University, Graduate School of Medicine Nagoya Japan; ^2^ Institute for Medical Science of Aging Aichi Medical University Nagakute Japan; ^3^ Moriyama General Mental Hospital Nagoya Japan; ^4^ Okehazama Hospital Fujita Mental Care Center Toyoake Japan; ^5^ Aichi Psychiatric Medical Center Nagoya Japan

**Keywords:** Alzheimer disease, delusion, Lewy body disease, parkinsonism, psychosis

## Abstract

**Aim:**

The pathological criteria from the fourth Consortium on Dementia With Lewy bodies (DLB) in psychiatric cohorts has not been validated. Also, the pathological differences in prodromal DLB subtypes remain unclear. This study aimed to elucidate the clinicopathological features of patients with DLB in psychiatric hospitals.

**Methods:**

Of 165 autopsied cases, patients who developed psychiatric symptoms at 50 years or older were investigated based on the current criteria of DLB. Clinicopathological findings were compared among prodromal DLB subtypes.

**Results:**

Sixteen of 30 cases with DLB pathology had no parkinsonism, which represented diverse nigral neurodegeneration. Regarding the scheme to estimate the likelihood of DLB syndrome, the prevalence of core clinical features excluding rapid eye movement sleep behavior disorder and probable DLB diagnosis were significantly higher in the high‐likelihood group than in the low‐likelihood group. Regarding the prodromal DLB subtypes, mild cognitive impairment (MCI) onset was identified in 60%, psychiatric onset in 20%, delirium onset in 10%, and motor onset in 10% of cases, and the proportion of psychiatric onset or delirium onset was significantly higher compared with those without DLB pathology. Coexistence of MCI and psychiatric symptoms was observed in 41.6% of the MCI‐onset cases. Patients with psychiatric‐onset cases were significantly younger at the onset, with a longer disease duration than those with MCI‐onset cases. No differences were observed in other clinicopathological variables among the subtypes.

**Conclusion:**

The fourth Consortium pathological criteria for DLB were applicable in a psychiatric cohort. MCI‐onset cases in conjunction with core clinical features is the main prodromal DLB subtype, and four cases exhibited isolated psychiatric symptoms for long‐term duration.

Dementia with Lewy bodies (DLB) is the second most frequent neurodegenerative disease after Alzheimer disease (AD).^1^ Previously, the diagnostic detection rates for DLB in clinical practice were suboptimal^2^; hence, the diagnostic criteria were revised in 2017.^1^ Alzheimer‐type pathology is commonly observed in the brains of patients with DLB. The revised neuropathological criteria are based on refinement of the pathological evaluation for Alzheimer‐type pathology as well as Lewy‐related pathology such as additional neuropathological categories of amygdala‐predominant and olfactory bulb–only subtypes. Furthermore, a semiquantitative assessment of the degree of neuronal loss in the substantia nigra (SN) and a description of the presence or absence of parkinsonism were recommended. These revisions provide a framework for the clinicopathological investigation of Lewy‐related pathology in various clinical settings, regardless of the presence or absence of parkinsonism.

Identifying patients who are in the early predementia stages is important for early intervention. The diagnostic criteria for prodromal DLB were published in 2020.^3^ The criteria proposed three clinical subtypes: mild cognitive impairment with Lewy bodies (MCI‐LB), psychiatric‐onset DLB, and delirium‐onset prodromal DLB. However, there is limited information regarding the clinical presentations of psychiatric‐ and delirium‐onset subtypes compared with that of the MCI‐LB subtype.^4^, ^5^, ^6^, ^7^, ^8^

In this study, we applied the fourth Consortium on Dementia With Lewy Bodies (CDLB) neuropathological criteria^1^ to autopsied cases who presented with psychiatric symptoms and required hospitalization until death. The aim was to validate the use of the revised neuropathological criteria in a psychiatric cohort. We also investigated the potential clinicopathological differences according to initial presentation subtypes, with a focus on psychiatric‐onset prodromal DLB.^3^, ^8^

## Materials and Methods

### Patient identification and selection procedures

**Fig. 1:**
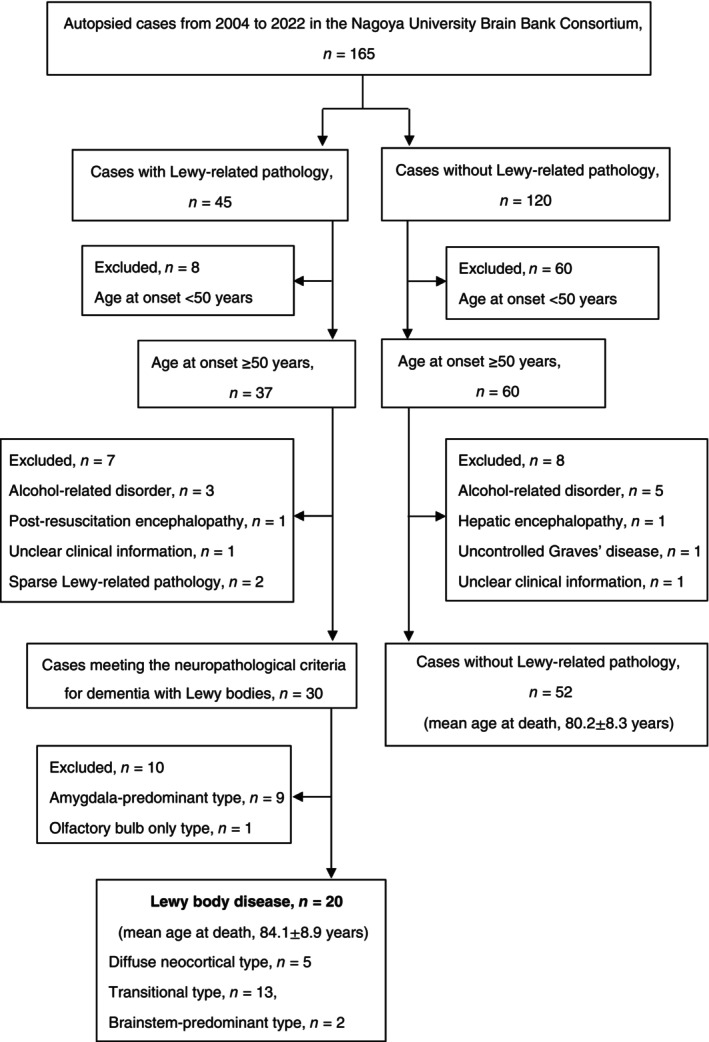
Flowchart of the study design. There was no difference in the mean age at death between 20 cases with Lewy body disease and 52 cases without Lewy‐related pathology.

The flowchart describes the study design (Fig. 1). All cases were selected from psychiatric hospitals (the Okehazama Hospital, Aichi Psychiatric Medical Center, and Moriyama General Mental Hospital in Japan), which comprise the Nagoya University Brain Bank Consortium. Between August 2004 and November 2022, 165 patients underwent neuropathological investigations. Based on the standardized neuropathological evaluations, 45 patients had Lewy‐related pathology.^1^ We focused on the relationship between late‐onset psychiatric manifestations and Lewy body (LB) pathology in this study; thus, we excluded eight cases with onset age younger than 50 years, and mainly included cases with schizophrenia or bipolar disorder.^8^, ^9^, ^10^ Owing to the difficulty in studying clinicopathological correlations, five cases were excluded. Additionally, two cases with sparse Lewy‐related pathology in the brain region failed to fulfill the fourth CDLB neuropathological criteria. Finally, data from 30 cases with DLB pathology were extracted for further clinicopathological investigations. Among the 60 cases with an onset age 50 years or older and no Lewy‐related pathology, 52 cases were analyzed after excluding eight cases (Fig. 1).

This study was approved by the Nagoya University Graduate School of Medicine ethics committee and complied with all provisions of the Declaration of Helsinki. Written informed consent was obtained from the patients' proxies, and their anonymity was retained.

### Clinical assessments and clinical diagnosis

We retrospectively reviewed the medical records and evaluated the clinical profiles of patients blinded to the neuropathological information. In most cases, the medical history was reconfirmed through interviews with the family and supplemented with medical records during the consent process for autopsy. All patients underwent head computed tomography. Dementia was defined as a progressive cognitive decline of sufficient magnitude to interfere with normal social or occupational functions, or with usual daily activities.^1^ The doses of antipsychotics and antiparkinsonian drugs from 1 year before death were calculated using chlorpromazine and levodopa equivalent dose.^11^, ^12^

The presence or absence of the core clinical features of DLB was evaluated for clinical diagnosis of probable DLB and possible DLB. According to the clinical criteria of the fourth CDLB,^1^ rapid eye movement sleep behavior disorder (RBD) was added as a fourth core clinical feature alongside three core clinical features such as cognitive fluctuations, recurrent visual hallucinations, and spontaneous parkinsonism. Because the previous core features were observed in the late stage of AD,^1^, ^13^ the occurrence of these core features within 3 years relative to the onset of dementia was regarded as their presence.^14^ As RBD episodes often become less vigorous or even quiescent with the progression of dementia,^1^ RBD was defined as a clinical history of repeated episodes of dream enactment behaviors.^15^

For the clinical subtypes of prodromal DLB, psychiatric symptoms or delirium are not uncommon during the MCI stage in patients with DLB.^16^, ^17^ In our cases, when both clinical features appeared in the context of progressive cognitive decline, they were classified as the MCI‐onset subtype owing to the unclear boundary among them. Psychiatric‐onset or delirium‐onset subtype was rigorously classified when each episode occurred in isolation and was confirmed to have no overt cognitive decline after full recovery from psychiatric symptoms or delirium.

### Neuropathologic techniques

All brains were removed after death and fixed in 20% formalin for at least 4 weeks. Macroscopic observations and dissection of specimens were then performed according to standard practice at the Institute for Medical Science of Aging, Aichi Medical University.^18^ Each cut and trimmed brain section was processed for paraffin embedding. In addition to hematoxylin–eosin (HE), Klüver‐Barrera, and Gallyas silver staining, the following primary antibodies were used for immunohistochemistry: anti‐phosphorylated‐tau (clone AT8; monoclonal mouse, 1:1000; Innogenetics), anti‐β‐amyloid (clone 6F/3D; monoclonal mouse, at 1:100; DAKO), phosphorylated 43‐kDa TAR DNA‐binding protein (pTDP‐43 ser409/410; polyclonal, 1:2500; CosmoBio), anti‐α‐synuclein (polyclonal rabbit, 1:1000; Sigma Aldrich) and anti‐phosphorylated α‐synuclein (pSyn#64, monoclonal mouse, 1:1000; Wako Pure Chemical Industries).

### Neuropathologic assessments

Each case assessor was blinded to the clinical data. Neurofibrillary tangles (NFTs), diffuse plaques, and neuritic plaques were assessed. Braak NFT staging,^19^, ^20^ Thal phase,^21^ and Consortium to Establish a Registry for AD (CERAD) neuritic plaque score^22^ were evaluated, and the AD neuropathological changes were categorized according to the ABC score from the National Institute on Aging‐Alzheimer's Association (NIA‐AA) criteria as follows: NIA‐AA none/low, NIA‐AA intermediate, and NIA‐AA high categories.^23^

According to the fourth CDLB neuropathological criteria,^1^ immunostaining with α‐synuclein and phosphorylated α‐synuclein were used to assess Lewy‐related pathology, such as LBs and the other α‐synuclein‐positive structures. Utilizing these stains, we determined the pathological subtypes of DLB: olfactory bulb–only, amygdala‐predominant, brainstem‐predominant Lewy body disease (LBD), limbic (transitional) LBD (TLBD), and diffuse neocortical LBD (DLBD) subtypes. Based on the pathological subtypes of DLB and ABC score of the NIA‐AA, we determined the DLB likelihood categories (Table 1).^1^ Neuronal loss in the SN was semiquantitatively assessed as none, mild, moderate, and severe.^1^, ^24^

**Table 1:** Application of the Fourth CDLB criteria to cases in a psychiatric cohort

		Alzheimer disease neuropathologic change		
		NIA‐AA none/low	NIA‐AA intermediate	NIA‐AA high
Lewy body pathology	Diffuse neocortical Lewy body disease	0	3, 100%, 2 (1.5–2)	2, 50%, 3 (3–3)
	Limbic (transitional) Lewy body disease	6, 67%, 1.5 (1–2.8)	5, 40%, 2 (1–2)	2, 50%, 2 (2–2)
	Brainstem‐predominant Lewy body disease	1, 0%, 1 (1–1)	1, 100%, 3 (3–3)	0
	Amygdala‐predominant	0	0	9, 22%, 0 (0–1)
	Olfactory bulb only	0	0	1, 0%, 0 (0–0)

Each box shows the number of cases, the ratio of parkinsonism during a lifetime, and the degree of neuronal loss in the substantia nigra (median [IQR]). Each category of likelihood scheme is color‐coded as high likelihood: white, intermediate likelihood: dark gray, low likelihood: light gray.

CDLB, Consortium on Dementia With Lewy Bodies; IQR, interquartile range; NIA‐AA, National Institute on Aging‐Alzheimer's association.

Argyrophilic grains (AGs) were assessed through Gallyas silver staining, and the Saito AG stage was evaluated accordingly.^25^ The severity of cerebral amyloid angiopathy (CAA), arteriolosclerosis, and large infarcts were assessed based on the previous studies.^26^, ^27^, ^28^, ^29^, ^30^

### Statistical analysis

Statistical analyses were performed using IBM SPSS Statistics (version 29). Fisher exact test was used to compare categorical variables. The Kruskal‐Wallis test was used to compare demographics and clinicopathological variables; the Bonferroni test was used as a *post hoc* analysis. In case #4, the age of onset was set at 58 years instead of the late 50s, for group comparison. The degree of neuronal loss in the SN, CAA, and arteriolosclerosis were quantified and compared as none = 0, mild = 1, moderate = 2, severe = 3. The severity of Lewy‐related pathology was quantified and compared in each region as none = 0, mild = 1, moderate = 2, severe = 3, and very severe = 4. CERAD scores were quantified and compared as none = 0, sparse = 1, moderate = 2, and frequent = 3. The score of Braak NFT staging of AD, Saito AG stage, and Thal phase corresponded to a figure of each stage or phase. All tests were two‐tailed, and *P*‐values <0.05 were considered statistically significant.

## Results

### Application of the DLB neuropathological criteria

For AD neuropathologic change, categories were assigned as none/low in seven cases, intermediate in nine cases, and high in 14 cases according to NIA‐AA guidelines (Table 1). Regarding LB pathology, there were five DLBD subtypes, 13 TLBD subtypes, and two brainstem‐predominant LBD (BLBD) subtypes. As additional categories, nine amygdala‐predominant subtypes and one olfactory bulb–only subtype were also identified. By applying the scheme for estimating the likelihood of the premortem DLB syndrome, cases were classified as high likelihood of DLB in nine cases, intermediate likelihood of DLB in seven cases, and low likelihood of DLB in 14 cases. A total of 14 cases had parkinsonism during their lifetime, with the degree of neuronal degeneration in the SN varying widely. Neuronal loss in the SN was semiquantitatively classified according to the DLB neuropathological criteria as none (*n* = 7, 23%), mild (*n* = 10, 33%), moderate (*n* = 8, 27%), and severe (*n* = 5, 17%). In accordance with the severity of nigral degeneration, the proportion of parkinsonism increased as follows: none (1/7, 14%), mild (4/10, 40%), moderate (5/8, 63%), and severe (4/5, 80%).

Clinical features were compared among the three likelihood groups (Table 2). Of these 30 cases with DLB pathology, 29 cases exhibited dementia. Only one case with BLBD, which was classified into the low likelihood of DLB group, exhibited no dementia. There were no differences in demographics, age at onset of dementia, and age at death among three likelihood groups. In contrast, the prevalence of three core clinical features, excluding RBD, and probable DLB diagnosis were significantly higher in the high‐likelihood group than in the low‐likelihood group, and the intermediate‐likelihood group was in between the high and low groups. In most cases with high or intermediate likelihood of DLB, the core clinical features of DLB occurred within 3 years of dementia onset.

**Table 2:** Comparison of clinicopathological features by the likelihood group in cases with DLB pathology

		High‐likelihood DLB (*n* = 9)	Intermediate‐likelihood DLB (*n* = 7)	Low‐likelihood DLB (*n* = 14)	*P‐*values
Clinical findings					
Sex (female/male)		4/5	4/3	5/9	0.729
Age at onset, median (IQR), years		78 (64.5–83)	77 (60–93)	77.5 (61.3–80.3)	0.974
Disease duration, median (IQR), years		5 (4–17.5)	6 (1–14)	5 (4–11)	0.95
Age at death, median (IQR), years		84 (81.5–91)	89 (78–94)	81.5 (69–88.3)	0.293
Age at onset of dementia, median (IQR), years		80 (76–84.5)	80 (74–93)	79 (68.5–82)	0.676
Dosage of antipsychotics, chlorpromazine equivalent, median (IQR), mg		50 (19–175.8)	100 (37.9–150)	50 (0–156.3)	0.894
Dosage of antiparkinsonian drugs, levodopa equivalent, median (IQR), mg		0 (0–0)	0 (0–0)	0 (0–0)	0.941
Dementia, %		100	100	93	1
Clinical diagnosis	Probable DLB, %	78^†^	29	7	0.001*
	Possible DLB, %	11	43	14	0.305
Core clinical features of DLB (within 3 years relative to the onset of dementia)	Fluctuating cognition/alertness, %	56^†^	43	7	0.025*
	Visual hallucinations, %	67^†^	43	14	0.037*
	REM sleep behavior disorder, %	11	0	7	1
	Parkinsonism, %	78^†^	29	14	0.009*
Pathological findings					
Saito Argyrophilic grain stage, median (IQR)		0 (0–0)	0 (0–0)	0 (0–0)	0.392
Cerebral amyloid angiopathy score, median (IQR)		0 (0–1)	0 (0–1)	0.5 (0–1)	0.987
Arteriolosclerosis score, median (IQR)		0 (0–1)	1 (0–1)	0 (0–1)	0.729
Large infarct, %		0	0	0	1

^†^ *P* < 0.05 compared with the low‐likelihood group. Fisher exact test or Kruskal‐Wallis test were used for comparison. Bonferroni test was used for *post hoc* analysis.

Statistically significant *P*‐values (*P* < 0.05) are marked with asterisks.

DLB, dementia with Lewy bodies; IQR, interquartile range; REM, rapid eye movement.

### Additional pathological categories of the amygdala predominant or olfactory bulb–only subtypes

In the setting of advanced AD with Braak NFT stage V or VI, it was reported that approximately 20% of the cases had LBs relatively confined to the amygdala and olfactory bulb.^31^, ^32^ In this series, all cases with the amygdala‐predominant or olfactory bulb–only subtype had concurrent advanced AD pathology with Braak NFT stage V or VI. While LBs in the brainstem nuclei were not identified in those cases on HE staining, immunostaining for α‐synuclein occasionally revealed some positive structures. Semiquantitative gradings of neuronal loss in the SN were assigned as none (*n* = 7) and mild (*n* = 3). These pathological findings corresponded to AD with amygdala LBs, which is a distinct form of α‐synucleinopathy.^31^, ^32^ Regarding clinical features, progressive cognitive decline was predominantly observed without the core clinical features of DLB in the early stages, supporting the low likelihood of DLB, which is negatively correlated with DLB clinical syndrome. Amygdala‐predominant and olfactory bulb–only subtypes shared clinicopathological characteristics with AD rather than DLB.^33^

### Application of clinical criteria for diagnosis of prodromal DLB

As opposed to amygdala‐predominant and olfactory bulb–only subtypes, LBs in the brainstem nuclei were identified on HE staining in 20 cases of the other three subtypes. These neuropathological subtypes were originally defined as LBD.^34^ When the 20 cases with LBD were classified by initial manifestations (Table 3), psychiatric symptoms or delirium as subtypes of prodromal DLB were identified in four cases (20%) and two cases (10%), respectively. In two cases (10%), motor symptoms preceded the onset of dementia by a few years without obtaining a clinical diagnosis of Parkinson disease (PD) (motor‐onset subtype).^35^ The remaining 12 cases (60%) were accompanied by cognitive decline as early manifestations (MCI‐onset subtype). Additionally, five cases (41.6%) in the MCI‐onset subtype showed psychiatric symptoms before being diagnosed with dementia. When clinical variables were compared among the four groups, the onset age of the psychiatric‐onset subtype was significantly lower than that of the MCI‐onset subtype. The motor‐onset subtype was prescribed significantly higher doses of antiparkinsonian medications compared with other subtypes. There was no difference in age at death among the four subtypes, and the psychiatric‐onset subtype had a significantly longer disease duration than the MCI‐onset subtype. There were no differences in the onset age of dementia and pathological findings among the four subtypes. These clinicopathological findings suggest that DLB clinical syndrome primarily appeared when patients were in their 70s and 80s, regardless of prodromal DLB subtypes.

**Table 3:** Comparisons of clinicopathological findings in 20 LBD according to initial presentations

			Psychiatric onset (*n* = 4)	Delirium onset (*n* = 2)	MCI onset (*n* = 12)	Motor onset (*n* = 2)	*P‐*values
Clinical findings							
Sex (female/male)			2/2	0/2	8/4	0/2	0.128
Age at onset, median (IQR), years			57 (54.5–59.5)^†^	74.5 (71–78)	78.5 (73.8–86)	80.5 (78–83)	0.047*
Disease duration, median (IQR), years			29.5 (16.3–38.3)^†^	4.5 (4–5)	5.5 (3.3–9.8)	5.5 (2–9)	0.032*
Age at death, median (IQR), years			88.5 (72.8–93.8)	79 (75–83)	85.5 (78.3–91.5)	86 (80–92)	0.792
Age at onset of dementia, median (IQR), years			76 (81–85)	76 (72–80)	79.5 (74.5–86)	83 (80–86)	0.693
Dosage of antipsychotics, chlorpromazine equivalent, median (IQR), mg			100 (40.9–225)	57 (38, 76)	75 (0–187.9)	0 (0, 0)	0.271
Dosage of antiparkinsonian drugs, levodopa equivalent, median (IQR), mg			0 (0–0)	0 (0, 0)	0 (0–0)	275 (100, 450)^‡^	0.007
Dementia, %			75	100	100	100	0.4
Clinical diagnosis		Probable DLB, %	50	50	50	0	0.799
		Possible DLB, %	25	0	17	100	0.136
Core clinical features of DLB (within 3 years relative to the onset of dementia)		Fluctuating cognition/alertness, %	25	50	50	0	0.654
		Visual hallucinations, %	75	50	42	0	0.62
		REM sleep behavior disorder, %	0	0	8	0	1
		Parkinsonism, %	50	50	42	100	0.743
Pathological findings							
Lewy‐related pathology type		Diffuse neocortical, %	50	0	25	0	0.645
		Limbic (transitional), %	25	100	75	50	0.239
		Brainstem‐predominant, %	25	0	0	50	0.147
Severity of Lewy‐related pathology	Brainstem regions	IX‐X, median (IQR)	2.5 (2–3)	2.5 (2–3)	3 (3–3)	2.5 (2–3)	0.4
		LC, median (IQR)	2.5 (2–3)	3 (3–3)	3 (3–3)	2.5 (2–3)	0.317
		SN, median (IQR)	2 (1.3–2)	2.5 (2–3)	2.5 (1.3–3)	2 (2–2)	0.538
	Basal forebrain/limbic regions	NBM, median (IQR)	2.5 (1.3–3.8)	2.5 (2–3)	3 (3–3)	2 (1–3)	0.602
		Amygdala, median (IQR)	3.5 (2.3–4)	2.5 (2–3)	3 (3–4)	2 (1–3)	0.278
		Transentorhinal, median (IQR)	2.5 (1.3–3.8)	1.5 (1–2)	3 (2–3)	1 (0–2)	0.129
		Cingulate, median (IQR)	1.5 (0.3–2.8)	1.5 (1–2)	2 (2–3)	1.5 (1–2)	0.227
	Neocortical regions	Temporal, median (IQR)	1.5 (0.3–2.8)	0.5 (0–1)	1.5 (1–2)	0.5 (0–1)	0.266
		Frontal, median (IQR)	0, 1, 2	0.5 (0–1)	1 (0–3)	0.5 (0–1)	0.72
		Parietal, median (IQR)	1, 3	0.5 (0–1)	0 (0–1)	0.5 (0, 1)	0.494
Brain weight, median (IQR), g			1141 (1086–1198)	1216 (1154–1278)	1149 (1051.5–1226)	1346 (1182–1510)	0.38
The degree of neuronal loss in the SN, median (IQR)			1 (1–1.8)	1.5 (1–2)	2 (1.3–2.8)	3 (3–3)	0.098
Braak neurofibrillary tangle staging, median (IQR)			4 (1.8–4)	2 (2–2)	4.5 (3–5.8)	3.5 (3–4)	0.188
Thal phase, median (IQR)			4 (1–4.8)	3.5 (3–4)	3 (1.5–5)	2 (1–3)	0.758
CERAD neuritic plaque score, median (IQR)			2 (0.3–3)	1 (1–1)	2 (0.3–2.8)	1 (1–1)	0.709
Saito Argyrophilic grain stage, median (IQR)			0 (0–0)	0 (0–0)	0 (0–0)	0 (0–0)	0.704
Cerebral amyloid angiopathy score, median (IQR)			0.5 (0–1)	0 (0–0)	1 (0–1)	0 (0–0)	0.174
Arteriolosclerosis score, median (IQR)			0 (0–0.8)	0 (0–0)	0.5 (0–1)	1.5 (1–2)	0.107
Large infarct, %			0	0	0	0	1

^†^ *P* < 0.05 compared with the MCI‐onset group.

^‡^ *P* < 0.05 compared with other groups. The onset age was set at 58 years instead of late 50s for group comparison. Fisher exact test or Kruskal‐Wallis test was used. Bonferroni test was used for *post hoc* analysis. When the number of sample was ≤3, all data instead of median value are shown.

Statistically significant *P*‐values (*P* < 0.05) are marked with asterisks.

CERAD, Consortium to Establish a Registry for Alzheimer's Disease; DLB, dementia with Lewy bodies; IQR, interquartile range; LBD, Lewy body disease; LC, locus coeruleus; MCI, mild cognitive impairment; NBM, nucleus basalis of Meynert; SN, substantia nigra; IX, 9th cranial nerve nucleus; X, 10th cranial nerve nucleus.

### Case presentations of psychosis‐onset cases with LBD

**Fig. 2:**
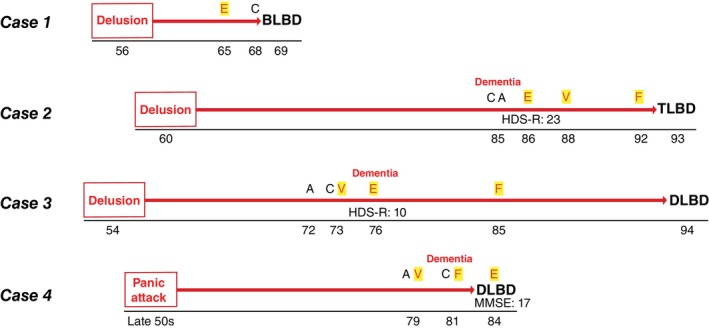
Clinical course of Lewy body disease in psychiatric‐onset subtype. Evolution of clinical features based on the age in four cases with psychiatric‐onset Lewy body disease. Patients' score of neuropsychological testing such as HDS‐R and MMSE, age at death, and pathological subtype of Lewy body disease at autopsy are also shown. Case 1: delusion—56; extrapyramidal symptoms (E)—65; cognitive decline (C)—68; and death—69. Autopsy showed BLBD. Case 2: delusion—60; auditory hallucinations (A)—85; dementia—85; HDS‐R: 23 points—86; E—86; visual hallucinations (V)—88; fluctuating cognition (F)—92; and death—93. Autopsy showed TLBD. Case 3: delusion—54; A—72; C—73; V—73; E—76; dementia—76; HDS‐R: 10 points—76; F—85; and death—94. Autopsy showed DLBD. Case 4: panic attack—50 years late; A—79; V—79; C—81; F—81; dementia—81; E—84; MMSE: 17 points—84; and death—84. Autopsy showed DLBD. BLBD, Brainstem‐predominant Lewy body disease; DLBD, Diffuse neocortical Lewy body disease; HDS‐R, Hasegawa Dementia Scale‐Revised; MMSE, Mini‐Mental State Examination; TLBD, Transitional Lewy body disease.

**Fig. 3:**
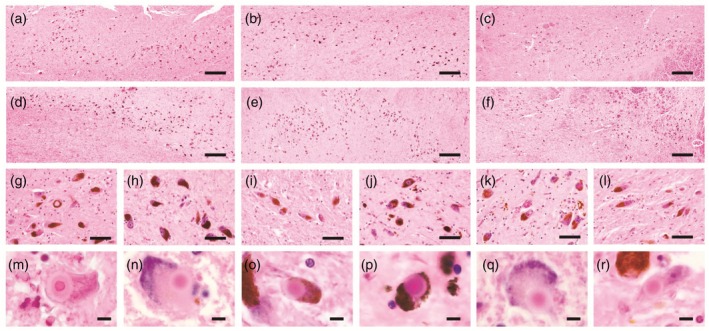
Nigral neurodegeneration in psychiatric‐onset or delirium‐onset cases. Pathological investigations revealed mild to moderate neurodegeneration in the substantia nigra and Lewy bodies on hematoxylin–eosin staining. Semiquantitative assessment of neuronal loss in the substantia nigra showed mild loss in Case 1 (a, g, m), Case 2 (b, h, n), Case 4 (d, j, p), and Case 5 (e, k, q), while moderate loss was observed in Case 3 (c, I, o) and Case 6 (f, l, r). Scale bars: 200 μm (a–f), 50 μm (g–l) and 5 μm (m–r).

The clinicopathological characteristics of four psychiatric‐onset cases and two delirium‐onset cases are summarized in Table 4.^36^, ^37^ In the psychiatric‐onset subtype, isolated psychiatric symptoms, such as anxiety and delusions, lasted for long periods without progressive cognitive decline (Fig. 2). During the long‐term isolated psychiatric presentations, visual hallucinations were not observed. Both psychiatric‐onset and delirium‐onset cases had mild to moderate neuronal loss in the SN and Lewy bodies (Fig. 3a–r), and the findings were consistent with a lack of spontaneous parkinsonism in the early stages. We reported the detailed clinical courses of three cases with late‐onset psychosis to highlight the clinical pictures of the psychiatric‐onset DLB subtype.

**Table 4:** Clinicopathological features of psychiatric‐onset and delirium‐onset DLB subtypes

			Case 1	Case 2	Case 3	Case 4	Case 5	Case 6
Clinical findings								
Initial clinical manifestation (age at onset, years)			Delusion (56)	Delusion (60)	Delusion (54)	Panic attack (late 50s)	Delirium (78)	Delirium (71)
Sex			Male	Female	Female	Male	Male	Male
Family history of psychiatric disease in first‐ or second‐degree relatives			Schizophrenia (brother)	Commit suicide (sister)	Not provided	Absent	Absent	Bipolar disorder (brother)
Physical medical condition at onset of initial psychiatric symptom			Diabetes Chronic renal failure Legal blindness	Age‐related hearing impairment	Graves disease (controlled)	Absent	Acute myocardial infarction Chronic renal failure	ANCA‐associated vasculitis Sjögren syndrome
Age at cognitive decline, years			68	85	73	81	80	72
Age at onset of extrapyramidal signs, years			65 Risperidone 2–2.5 mg/d for 9 years	86 Transient occurrence by aripiprazole 12 mg/d	76	84	82	72 Transient occurrence by olanzapine 5 mg/d
Age at death, years			69	93	94	84	83	75
Score of Hasegawa dementia rating scale‐revised, points (age at test, years)			NE	23 (86)	10 (76)	NE	21 (80)	10 (72)
Score of Mini‐Mental State Examination, points (age at test, years)			NE	NE	NE	17 (84)	22 (80)	NE
Dosage of antipsychotics, chlorpromazine equivalent, mg			250	150	50	37.9	37.9	75.8
Dosage of antiparkinsonian drugs, levodopa equivalent, mg			0	0	0	0	0	0
Pathological findings								
Lewy‐related pathology type			Brainstem‐predominant	Limbic (transitional)	Diffuse neocortical	Diffuse neocortical	Limbic (transitional)	Limbic (transitional)
Severity of Lewy‐related pathology	Brainstem regions	IX‐X	2	3	2	3	2	3
		LC	2	3	2	3	3	3
		SN	1	2	2	2	2	3
	Basal forebrain/limbic regions	NBM	1	3	2	4	2	3
		Amygdala	2	3	4	4	3	2
		Transentorhinal	1	2	3	4	1	2
		Cingulate	0	1	2	3	1	2
	Neocortical regions	Temporal	0	1	2	3	0	1
		Frontal	0	1	NE	2	0	1
		Parietal	NE	1	NE	3	0	1
Brain weight, g			1246	1044	1100	1182	1278	1154
Degree of neuronal loss in the SN			Mild	Mild	Moderate	Mild	Mild	Moderate
Braak neurofibrillary tangle staging			1	4	4	4	2	2
Thal phase			0	4	4	5	4	3
CERAD neuritic plaque score			0	1	3	3	1	1
AD neuropathologic change (NIA‐AA guidelines)			Not	Intermediate	Intermediate	Intermediate	Low	Low
Likelihood of DLB pathology			Low	Intermediate	High	High	High	High
Saito Argyrophilic grain stage			0	0	0	0	0	0
Cerebral amyloid angiopathy score			0	1	0	1	0	0
Arteriolosclerosis score			0	1	0	0	0	0
Large infarct			None	None	None	None	None	None

AD, Alzheimer disease; ANCA, anti‐neutrophil cytoplasmic antibody; CERAD, Consortium to Establish a Registry for Alzheimer's Disease; DLB, dementia with Lewy bodies; LC, locus coeruleus; NBM, nucleus basalis of Meynert; NE, not evaluated by α‐synuclein immunostaining; NIA‐AA, National Institute on Aging in collaboration with the Alzheimer's Association; PD, Parkinson's disease; SN, substantia nigra; IX, 9th cranial nerve nucleus; X, 10th cranial nerve nucleus.

#### Case 1

A 54‐year‐old man began dialysis for diabetic renal failure and retired from his job as a truck driver. At the age of 56 years, he was diagnosed as legally blind due to diabetic retinopathy. Delusions of poisoning and persecution emerged. He heard human voices in his head telling him that men placed a bomb in his stomach, and that they would shoot him to death with guns. He insisted that poisoned water and dishes caused his blindness. He had difficulty in living at home owing to his refusal to eat. He was diagnosed with late‐onset schizophrenia.^38^ After treatment with risperidone (2 mg/day) for half a year during hospitalization, his delusions became unremarkable. However, at the age of 57 years, his delusions relapsed with tapering of the medication, and hospital treatment for delusions and abnormal behaviors was required until death. At the age of 65 years, extrapyramidal symptoms became evident. He exhibited flexed posture, sialorrhea, and unstable gait, indicating neuroleptics‐induced extrapyramidal signs (risperidone 2–2.5 mg/day for 9 years). At the age of 68 years, he presented with cognitive decline. At the age of 69 years, he died of repeated aspiration pneumonia.

**Fig. 4:**
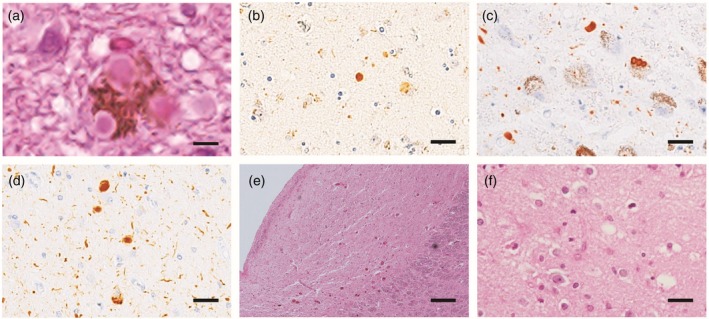
Microscopic findings in the amygdala and locus coeruleus in cases with late‐onset psychosis. Brainstem‐type Lewy bodies in the locus coeruleus (a) and cortical Lewy–related pathology in the amygdala (b) were identified in Case 1. Abundant Lewy–related pathology was observed in the locus coeruleus (c) and amygdala (d) in Case 2. Severe neuronal loss in the locus coeruleus (e) and spongiform changes with astrogliosis in the amygdala (f) were observed in Case 3. (a, e, f): hematoxylin–eosin staining; (b–d): immunohistochemical staining of phosphorylated alpha‐synuclein. Scale bars: 5 μm (a), 50μm (b–d, f), and 200 μm (e).

Neuropathological investigation revealed BLBD without AD neuropathologic change, which was consistent with a lack of dementia. Isolated delusion in this case may support the previous finding that the accumulation of LBs in the dorsal raphe nucleus was clinicopathologically associated with delusions in LBD.^39^ Mild neuronal loss in the SN was observed (Fig. 3a, g). Long‐term administration of neuroleptics may unmask dopaminergic vulnerability due to nigrostriatal degeneration and induce extrapyramidal signs. Both brainstem‐type LBs and cortical Lewy‐related pathology were identified in the locus coeruleus and the amygdala, respectively (Fig. 4a, b).

#### Case 2

A 60‐year‐old woman moved in with her family and developed persecutory delusions toward the residents on the upper floors for approximately 1 year. She insisted that the residents eavesdropped on her and talked about her, and often quarreled with them. At the age of 65 years, she lived alone after the death of her spouse and began to blame her eldest daughter for stealing her money. At the age of 75 years, she moved out because her delusional behaviors caused trouble with the other residents in the apartment. Her family persuaded her to visit a psychiatrist, but the patient refused. At the age of 85 years, she was admitted to a nursing home owing to the death of her youngest daughter, who took care of her. The patient exhibited auditory hallucinations, including construction‐related noise on the upper floor that did not exist. At the age of 86 years, her delusions and explosive temper became exacerbated, and she claimed that people were taking stimulant drugs and lurking in the ceiling to kill her. She consulted a doctor and scored 23 points on the Hasegawa Dementia Scale‐Revised (HDS‐R) (cutoff level: 20/30 points). Drug treatment with aripiprazole (12 mg/day) induced resting tremor, and thereafter she refused to visit a hospital. At the age of 88 years, she was hospitalized for exacerbating delusions. That same year, she developed visual hallucinations of crashing airplanes and people walking by. At the age of 93 years, she died of pneumonia with chronic heart failure.

Neuropathological investigations revealed TLBD with intermediate AD pathology, which was consistent with incident dementia in the late stage. Although transient tremor was induced by the administration of aripiprazole, mild neuronal loss in the SN supported the absence of spontaneous parkinsonism (Fig. 3b, h). More abundant LB pathology was observed in the locus coeruleus and amygdala than the SN (Fig. 4c, d).

#### Case 3

A 54‐year‐old woman presented with delusions of poverty and auditory hallucinations. She claimed to have “heard a stranger's voice,” and asked staff to hand over a box of tissues as she lives “in poverty,” despite having no financial problems. She was hospitalized with a clinical diagnosis of late‐onset schizophrenia.^29^ Although her psychosis improved temporarily, the patient relapsed at the age of 55 years, requiring hospitalization. After discharge at the age of 56 years, the patient's psychosis went into remission without pharmacotherapy, and she could perform her housework. However, at the age of 66 years, delusions of guilt and self‐injury behavior emerged, and she visited a psychiatrist. At the age of 67 years, her psychosis was in a state of remission, and she stopped attending regular hospital visits. At the age of 72 years, the patient was hospitalized due to prominent late‐night prowling dominated by overt auditory hallucinations. The patient reported having to search for her brother because he had radioed her that he was in financial need owing to his illness. At the age of 73 years, she developed memory disturbance and visual hallucinations. She also reported that strangers came into her room and doused hydrochloric acid over her head. At the age of 76 years, she presented with bradykinesia. Her psychotic symptoms almost vanished, and her HDS‐R score was 10/30. At the age of 94, she died of old age.

Neuropathological investigation revealed DLBD with intermediate AD pathology, which was consistent with common presentations of DLB after the age of 73 years. Moderate neuronal loss in the SN suggested mild parkinsonism (Fig. 3c, i), and severe neuronal loss in the locus coeruleus, and spongiform changes with astrogliosis in the amygdala were observed (Fig. 4e, f).

### Psychiatric‐onset cases with no Lewy‐related pathology

Among the 52 cases with no Lewy‐related pathology, four (7.6%) presented with psychiatric onset, while none exhibited delirium onset. The pathological diagnoses of the psychiatric‐onset cases included intermediate AD neuropathologic changes in two cases, primary age‐related tauopathy^40^ with Saito AG stage I in one case, and progressive supranuclear palsy in one case.^41^ In addition to LBD, these pathological findings of the psychiatric‐onset cases were compatible with those reported in previous clinicopathological studies.^41^, ^42^, ^43^ Compared with the group, the LBD group demonstrated a significantly higher proportion of psychiatric or delirium‐onset cases (*P* = 0.023), supporting a potential association between these initial symptoms and LBD pathology.

## Discussion

### Application of clinical subtypes according to initial presentations

In 1990, Kosaka clinicopathologically reviewed autopsied cases with DLBD in Japan.^44^ In 29 cases with DLBD whose age at onset was ≥50 years (mean age at onset: 68.3 years), initial symptoms were characterized by memory disturbance in 16 cases (55.1%), psychotic state in six cases (20.6%), parkinsonism in four cases (13.7%), and autonomic failure in three cases (10.3%). In many cases, parkinsonism was markedly observed in the later stage, but eight of 29 cases exhibited no parkinsonism even in the terminal stage. Autopsied cases at psychiatric hospitals included a 75‐year‐old man with a clinical diagnosis of senile psychosis without parkinsonism and a 65‐year‐old man with a clinical diagnosis of schizophrenia. There was no description regarding delirium as an initial manifestation.

Mellergaard *et al*. investigated the prodromal clinical characteristics of 166 patients with DLB in a memory clinic.^16^ Various noncognitive manifestations simultaneously occurred during the prodromal phase in most cases: psychiatric symptoms and delirium/acute confusional episodes were observed before DLB diagnosis in 81% and 33%, respectively. In contrast, 91(55%) of 166 patients with DLB showed one isolated initial symptom i.e. cognitive decline in 33%, psychiatric symptoms in 6%, and delirium/acute confusional episodes in 2%. Jicha *et al*. compared clinical profiles during MCI stage between autopsy‐confirmed DLB and AD.^17^ Among nine patients with DLB, four (44%) had provoked hallucinations/delirium at the MCI stage, whereas none of the 12 patients with AD exhibited these symptoms. Previous clinical studies also reported frequent coexistence of MCI and psychiatric symptoms at prodromal DLB state.^16^, ^17^, ^45^, ^46^, ^47^ In our series, 41.6% of those with the MCI‐LB subtype had psychiatric symptoms before dementia diagnosis. We rigorously categorized cases into psychiatric‐onset or delirium‐onset subtype based on domination by a single isolated manifestation without progressive cognitive decline. Finally, 30% of patients with LBD were classified into these subtypes. Moreover, the motor‐onset subtype was identified in a small number of patients (10%). The distinction in the proportion of subtypes was presumed to be due to different referral reasons or selection bias in autopsy cohorts.^48^, ^49^ Our cases were predominantly classified as MCI subtype, and the overlap of clinical presentations among subtypes was observed.

### Long‐term isolated psychiatric presentations in psychiatric‐onset DLB

De Pablo‐Fernández *et al*. clinicopathologically investigated the clinical subtypes of 111 autopsied cases with PD from the Queen Square Brain Bank.^50^ Based on a subtyping system using four clinical features (motor severity, cognitive impairment, RBD, and dysautonomia) from the Parkinson's Progression Markers Initiative data,^51^ patients were classified into three subtypes i.e. mild motor–predominant subtype (48.7%), diffuse malignant subtype (16.2%), and intermediate subtype for patients who failed to meet criteria for the other subtypes (35.1%). The three subtypes showed different rates of deterioration; however, there were no differences in mean age at death among them. The mild motor–predominant subtype was characterized by a young age at onset (mean age at diagnosis: 58.2 years) and slow progression, with a long duration before reaching the terminal stages of the disease. These patients exhibited a more classic PD phenotype, with tremor‐dominant parkinsonism, good levodopa responsiveness, and mild nonmotor symptoms. A subset of patients in this group were presumed to present with isolated parkinsonism for a long period. In contrast, the diffuse malignant subtype was characterized by an older age at onset (mean age at diagnosis: 70.3 years), less tremor‐dominant parkinsonism, less levodopa responsiveness, and severe nonmotor symptoms, which suggested an overlap with the clinical features of DLB. Considering no difference in neuropathological findings among the three groups, this study emphasized the importance of age at diagnosis as determinants of clinical subtypes.

In our series, parkinsonism was identified in almost half of cases with LB pathology, and the clinical findings were consistent with neuronal numbers in the SN. In contrast, all cases exhibited both cognitive decline and psychiatric symptoms that required in‐hospital treatment. Regardless of the clinical subtypes determined based on the initial symptoms, there were no differences in mean age at death and neuropathological findings based on standardized assessments. Most of our cases had an older age at onset, less tremor‐dominant parkinsonism, less levodopa responsiveness, and severe nonmotor symptoms such as dementia; these features overlapped with the clinical features of diffuse malignant subtype rather than mild motor–predominant subtype. Only the psychiatric‐onset subtype was characterized by a young age at onset (mean age at onset: 57 years) with psychiatric symptoms and a slow progression with a long duration before reaching the dementia stages. As the onset age of cognitive decline, the central feature of DLB, did not differ among the subtypes, most patients presented with DLB clinical syndrome in their 70s and 80s, regardless of prodromal DLB subtypes. Overall, the age at initial onset in psychiatric cohorts may be a key determinant of long‐term isolated psychiatric manifestation. Recent clinical reports supported the existence of long‐term disease nature in a subset of patients with psychiatric‐onset DLB.^8^, ^52^, ^53^

### Predicting factors for phenoconversion

A single isolated manifestation is observed in patients with isolated RBD or pure autonomic failure (PAF). Longitudinal follow‐up studies of these patients revealed predicting factors for phenoconversion, which may provide better insights into understanding the overall clinical courses in patients with LBD.

In men with isolated RBD, recurrent nocturnal dream enactment behaviors with injuries typically emerged in their 50s and 60s.^54^ To date, three autopsied cases of isolated RBD have been reported, with onset ages of 57, 64, and 67 years.^55^, ^56^, ^57^ Despite varying disease durations (2, 15, and 20 years), all cases had BLBD. A multicenter follow‐up study revealed that clinical measures at baseline significantly predicted subsequent phenoconversion.^58^ Converters showed significantly higher ages and greater abnormality on quantitative motor testing, neuropsychological examination, and dysautonomia at baseline. Therefore, typical patients with isolated RBD without any abnormal measures beyond polysomnographic recording tend to have long‐term stable conditions for several years or decades. These characteristics overlap with those of the classic PD phenotype regarding the single isolated clinical feature, despite demonstrating different predominant clinical features (RBD vs parkinsonism).

In a prospective cohort in the United States, Kaufmann *et al*. reported on the natural history of PAF until phenoconversion.^59^ The mean age of onset for neurogenic orthostatic hypotension was 63 ± 14 years. The clinical period of isolated dysautonomia was longer when the age at onset was younger. In six patients who developed PAF at 60 years or younger, the mean clinical duration of isolated dysautonomia was 18.1 years until conversion. In 13 patients who developed PAF at older than 60 years, the mean clinical duration of isolated dysautonomia was 8.4 years until conversion. In this cohort, probable RBD diagnosis was strongly associated with phenoconversion. Deficits in olfaction, subtle motor signs, and greater dysautonomia at baseline also predicted phenoconversion.

Although the predominant clinical features were different, a single isolated clinical feature may be a key factor for a long‐term stable condition without phenoconversion. In our study, cases with the psychiatric‐onset subtype mainly presented with delusion and anxiety as a single isolated clinical feature. These psychiatric symptoms corresponded to the supportive clinical features of DLB such as olfactory impairment and constipation. The supportive clinical features often preceded the onset of DLB by decades. In contrast, the core clinical features of DLB mainly appeared in patients in their 70s and 80s in the context of cognitive decline. The core features, especially visual hallucinations, occurred within 3 years relative to the onset of dementia. Therefore, in the management of psychiatric symptoms, the identification of recurrent visual hallucinations may be useful as an important predictor for the development of DLB.

### Limitations

The study has some limitations; first, clinical symptoms and signs regarding DLB features were not systematically evaluated based on standardized methods.^15^, ^60^, ^61^, ^62^ All patients, however, had detailed clinical charts, which were recorded by physicians, nurses, occupational therapists, and social workers. These medical records were sufficient to assess clinical features. For RBD episodes, the retrospective analysis of medical records may have underestimated its prevalence owing to less symptomatic nature in accordance with disease progression. Second, the sample size was small, especially of the psychiatric‐onset subtype. Third, multifaceted assessments were not systematically conducted. Multidisciplinary approaches integrating comprehensive psychosocial evaluations with biological investigations are crucial for a better understanding of the development of psychiatric symptoms.

## Conclusion and Future Directions

1. The frameworks of the fourth CDLB criteria are applicable to psychiatric patients. The revised pathological scheme, such as additional LB categories (the amygdala‐predominant or olfactory bulb–only subtypes) and semiquantitative assessment of nigral neurodegeneration, is useful for estimating the likelihood of the premortem DLB syndrome.
2. As the main prodromal DLB subtype, MCI in conjunction with core clinical features of DLB appeared in patients in their 70s and 80s. In the context of progressive cognitive decline during the predementia stage, psychiatric symptoms concurrently occurred in 41.6% of the MCI‐onset cases.
3. A subset of LBD cases developed isolated psychiatric symptoms in their 50s and 60s. Considering the long‐term period from initial psychiatric manifestations to the onset of dementia, it might be difficult to determine the timing of LBD onset. Further clinicopathological studies focusing on early LB pathological changes with controlled samples are essential.
4. Given the development of biomarkers, particularly using real‐time quaking‐induced conversion assays,^63^, ^64^, ^65^ a biology‐based approach to defining the disease^66^ may encourage reorganizing the phenotypic variability of LBD, with a focus on psychiatric‐onset prodromal DLB.

## Disclosure statement

Norio Ozaki and Masashi Ikeda are editorial board members of *Psychiatry and Clinical Neurosciences* and co‐authors of this article. To minimize bias, they were excluded from all editorial decision‐making related to the acceptance of this article for publication. The other authors declare no conflicts of interest in association with the present research.

## Author contributions

Concept and design of the study: K.T., H.F., Y.T., S.A., M.Y., S.I., Y.I., M.I. Acquisition and analysis of data: K.T., H.F., Y.T., S.A., H.S., C.H., A.M., N.O., M.Y., S.I., Y.I., M.I. Drafting the manuscript and figure: K.T., H.F., Y.I. Critical revision: H.F., Y.T., S.A., H.S., C.H., A.M., N.O., M.Y., S.I., Y.I., M.I.
